# Contactless: a new personalised telehealth model in chronic pediatric diseases and disability during the COVID-19 era

**DOI:** 10.1186/s13052-021-00975-z

**Published:** 2021-02-12

**Authors:** Eugenio Mercuri, Giuseppe Zampino, Alisha Morsella, Marika Pane, Roberta Onesimo, Carmen Angioletti, Piero Valentini, Claudia Rendeli, Antonio Ruggiero, Lorenzo Nanni, Antonio Chiaretti, Giovanni Vento, David Korn, Emilio Meneschincheri, Paolo Sergi, Giovanni Scambia, Walter Ricciardi, Andrea Cambieri, Antonio Giulio De Belvis

**Affiliations:** 1 Pediatric Neurology Unit, Fondazione Policlinico A Gemelli –IRCCS, Università Cattolica del Sacro Cuore, Largo Gemelli 8, 00168 Rome, Italy; 2 Centro Clinico Nemo, Fondazione Policlinico Universitario Agostino Gemelli –IRCCS, Rome, Italy; 3 Department of Woman and Child Health and Public Health, Public Health Area, Fondazione Policlinico Universitario Agostino Gemelli –IRCCS, Rome, Italy; 4 Pediatric Unit, Fondazione Policlinico Universitario Agostino Gemelli –IRCCS, Rome, Italy; 5 Rare Diseases Unit, Fondazione Policlinico Universitario Agostino Gemelli –IRCCS, Rome, Italy; 6 Critical Pathways and Outcomes Evaluation Unit, Fondazione Policlinico Universitario Agostino Gemelli –IRCCS, Rome, Italy; 7 Spina Bifida Unit, Fondazione Policlinico Universitario Agostino Gemelli -IRCCS, Rome, Italy; 8 Pediatric Oncology Unit, Fondazione Policlinico Universitario Agostino Gemelli –IRCCS, Rome, Italy; 9 Pediatric Surgery Unit, Fondazione Policlinico Universitario Agostino Gemelli –IRCCS, Rome, Italy; 10 Division of Neonatology, Fondazione Policlinico Universitario Agostino Gemelli –IRCCS, Rome, Italy; 11 ICT management Fondazione Policlinico Universitario Agostino Gemelli –IRCCS, Rome, Italy; 12 Ginecology Oncology Unit, Fondazione Policlinico Universitario Agostino Gemelli –IRCCS, Rome, Italy; 13 Section of Hygiene, Fondazione Policlinico Universitario Agostino Gemelli –IRCCS, Rome, Italy; 14 Healthcare Management - Fondazione Policlinico Universitario Agostino Gemelli -IRCCS, Rome, Italy

## Abstract

**Background:**

Suspending ordinary care activities during the COVID-19 pandemic made it necessary to find alternative routes to comply with care recommendations not only for acute health needs but also for patients requiring follow-up and multidisciplinary visits. We present the ‘Contactless’ model, a comprehensive operational tool including a plurality of services delivered remotely, structured according to a complexity gradient, aimed to cover diagnostic procedures and monitor disease progression in chronic pediatric patients.

**Methods:**

A multidisciplinary and multiprofessional project team was recruited, in collaboration with patients’ associations, to map a panel of available Evidence-Based solutions and address individual needs in full respect of the concept of personalized medicine. The solutions include a number of services from videoconsultations to more structure videotraining sessions.

**Results:**

A modular framework made up of four three Macro-levels of complexity - Contactless Basic, Intermediate and Advanced - was displayed as an incremental set of services and operational planning establishing each phase, from factors influencing eligibility to the delivery of the most accurate and complex levels of care.

**Conclusion:**

The multimodal, multidisciplinary ‘Contactless’ model allowed the inclusion of all Units of our Pediatric Department and families with children with disability or complex chronic conditions. The strengths of this project rely on its replicability outside of pediatrics and in the limited resources needed to practically impact patients, caregivers and professionals involved in the process of care. Its implementation in the future may contribute to reduce the duration of hospital admissions, money and parental absence from work.

**Supplementary Information:**

The online version contains supplementary material available at 10.1186/s13052-021-00975-z.

## Introduction

The COVID-19 pandemic has posed an unprecedented challenge to health systems and activated a timely response on behalf of governments and healthcare facilities. Many nations worldwide have responded by promptly reorganizing hospitals. Most resources were reserved for emergency care and for the development of COVID-19 units while, at the same time, limiting access to healthcare facilities for routine or elective activities, reducing the risk of spreading COVID-19 [1]. During the first months of the pandemic, severely affected countries had no choice but to cancel all elective surgeries, follow-up appointments, rehabilitation services [2]. This pressurized physicians and patients to find alternative ways to comply with care recommendations, especially in patients with chronic diseases, who require regular follow up assessments and of a multidisciplinary approach to prevent complications caused by undermanagement [3]. The challenge was to find alternative ways to continue treatment while maintaining safety measures. As an immediate response, physicians found themselves obliged to find quick-fixes to assist their patients remotely using informal telephone consultations and video-calls on disparate Apps [4]. This however could only be seen as an ‘emergency’ short term solution as it could not replace the quality of care previously provided and was not fully compliant with the protection of privacy. As the weeks passed, innovative digital devices [5], which had been slowly making their way into the market before the pandemic, paved the road for more advanced forms of remote care and surveillance and, as emergencies often do, the shift to telemedicine became one of the priority of the COVID-19 era [6].

Telehealth, a term used interchangeably with telemedicine, has been defined as the use of medical information that is exchanged from one site to another through electronic communication to improve a patient’s health [7]. This generally implies the use of multimodal devices that cannot however be easily made available for immediate use for large cohorts of patients. This appears to be particularly true for those pediatric patients in need of a multidisciplinary approach that also includes rehabilitation, or other aspects of care that are not always covered by the available devices. There has therefore been the need to identify a new, easily available approach, that would include the possibility to continue assessments and training sessions normally used in the routine clinical assessments as part of standards of care to restore care continuity to those who have been deprived of it. In this context, our Pediatric Department has been rapidly reorganizing the provision of its services to ensure uninterrupted follow-up and treatment, despite the trying circumstances. Our Department includes services focused on the care of chronic conditions such as Rare Diseases, Pediatric neurology, Neuromuscular and Spina Bifida Units, along with general pediatric oncology and pediatric surgery. Most patients require a broad range of services, from simple health-status checks to high complexity levels of care, and support for families who play a vital role in disease management and surveillance.

The purpose of the Contactless Project was to fill the gaps between simple video-consultations and more sophisticated telemedicine requiring the use of multimodal devices by identifying a number of intermediate steps. These included video tutorials and questionnaires delivered through remote interaction between the young patients and their parents or guardians to ensure continuity of care at different levels of complexity.

This article aims to describe a comprehensive organizational model that can provide the multiplicity of pediatric services through remote interaction with the families utilizing appropriate technologies with full respect for their privacy. We also report the development of this process, from design and personalization of the service to needs assessment and implementation planning.

## Methods

A multidisciplinary and multiprofessional project team of administrative and clinical Units of our institution, was recruited combining the expertise of the different FPG-IRCCS’ Pediatric Units, Information Communucation Technology Directorate, Data Protection Office, Marketing Unit, and Critical Pathways and Outcome Evaluation Unit which also provided organizational and managerial support.

A literature search mapped Evidence-Based services currently available in Telehealth in pediatric care. In the absence of structured models applicable to our needs, the Pediatric Department’s clinicians conducted a needs assessment to estimate which aspects of care to prioritize, and the volumes of the target population eligible to the services. A comprehensive analysis of the number and type of evaluations performed in the previous 3 years was performed. The needs assessment analysis led to an estimated volume of 1366 patients per month across the different pediatric specialties.

The second phase identified which tools were needed and the best modalities to provide the services. While some services could be delivered via videoconferences, more complex assessments, monitoring or interventions would require the involvement of the families to perform some activities. Each pediatric service established the most relevant needs and possible suggestions to ‘translate’ what normally used in a clinical setting into new tools that would enable families to perform these activities, with the help of videotutorials, questionnaires or interactive videotraining sessions. While we tried to develop tools that could be used across different disease groups, we also tried to recognize and address the specificity of the needs of individual groups in full respect of the concept of personalized medicine. The interaction between clinicians and managerial members of the team helped to further define which services could be delivered remotely. The Directorate was entitled to safeguard patients’ and providers’ privacy and set-up a direct connection of the services with the hospital’s Informative System, to document telemedicine visits in the hospital’s Electronic Medical Records. During this process, remote training programs for clinicians and families were defined along with quality assurance evaluation systems and Impact Measurement tools. Several key process indicators will be adopted to monitor the model and verify the effects of the introduction of the case manager (Table 1).

**Table 1:** Responsibility and activity Matrix for the ‘Contactless Basic’ package

Activities	Dedicated figure	Figure to be engaged	Figure to be informed
Training the Case-manager	Complex Operative Unit of Critical Pathways and Outcome Evaluation/ ICT Directorate	Case manager	
Discharging patients and establishing eligibility to the ‘Contactless Basic’ Package	Attending physician	Patient	Case Manager
• Verifying the technological requirements necessary for the service • Providing information regarding the administrative aspects of the service • Collecting consent modules for data processing • Instructing parents or guardians on the use ‘Contactless Basic’ • Inserting appointments in the virtual agenda • Requesting necessary documentation and verifying the documentation received	Case Manager	Patient	Attending physician
• Paying (if applicable) and sending prescriptions	Patient	Case Manager	Attending physician
• Verifying payments, if applicable • Uploading clinical documentation received • Solving of technical issues	Case Manager	Patient	Attending physician
Preparing medical reports according to norms and regulations/ designing the treatment plan and setting up subsequent appointments	Attending physician	Patient	Case Manager
Uploading the medical report through the App	Patient	Case Manager	Attending physician

## Results

A modular framework was developed to display an incremental set of services, establishing each step, from the identification of patients to the delivery of the most accurate and complex level of care. Eligibility to the service was established by examining all pediatric patients requiring regular follow-up. Eligibility for the service was defined according to several influencing factors. We prioritized patients enrolled in long-term follow-up, at risk of complications, needing regular multidisciplinary assessments as suggested by available care recommendations. Other criteria to define priority were lack of adequate alternative territorial assistance and the distance from our centre, as transport could constitute a significant burden on the family in terms of time, cost and safety, at a time when social distancing might be recommended for a long time. We also preliminarily established whether familes could provide adequate technological resources for the chosen service (e.g. smartphone, home network, computer).

**Fig. 1:**
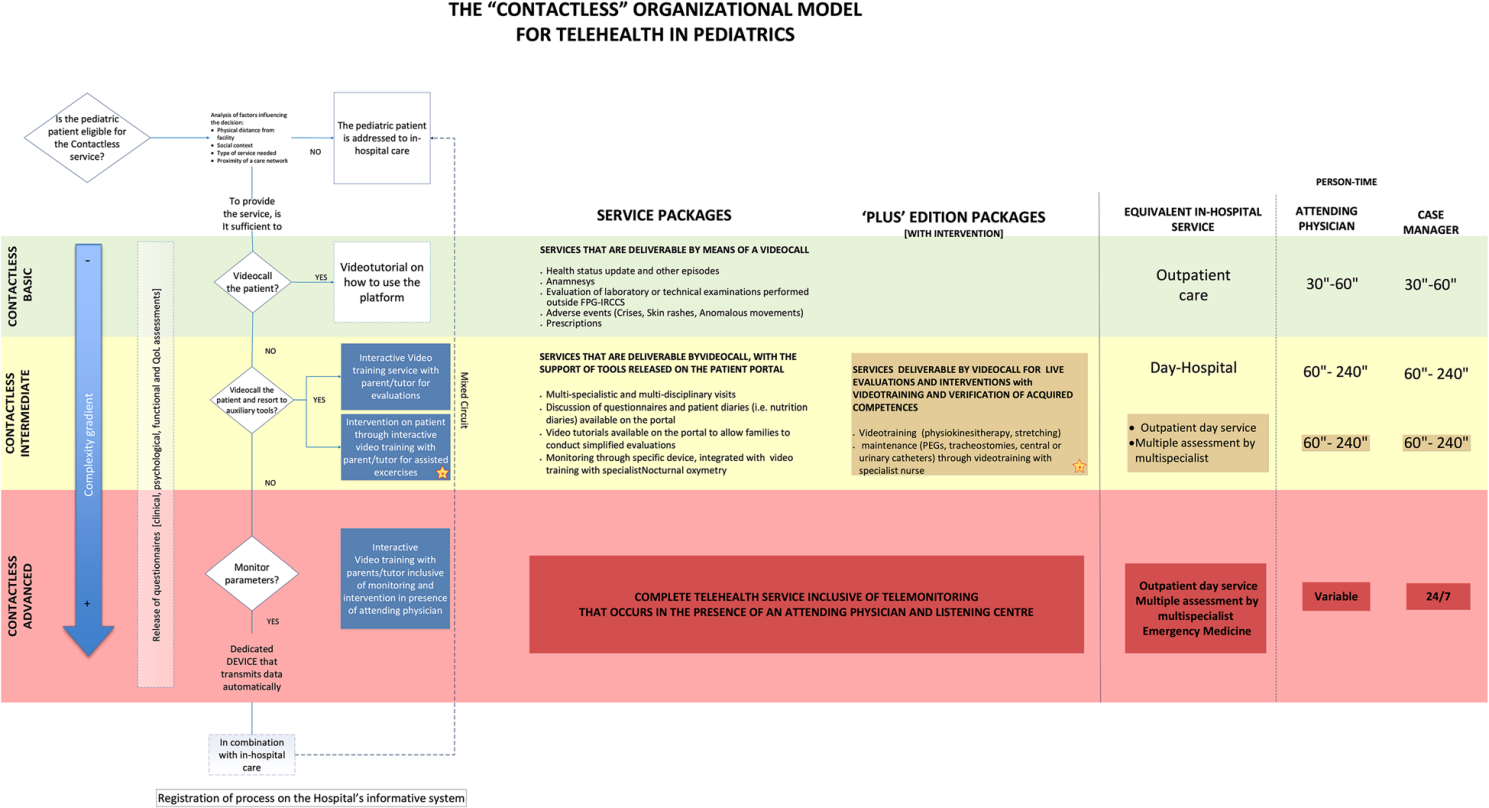
The "Contactless" organizational model for telehealth in pediatrics

What follows is the description of the services included in Contactless from lowest to highest complexity (Fig. 1).

### Basic level

The ‘Basic’ set of services is addressed to all patients for whom a videocall consultation is sufficient. This is the case of health-status updates, communication of medical history, discussion of adverse events (e.g. crises, skin rashes or abnormal movements) viewable on video by an examiner. This also includes assessment and discussion of lab tests or other results performed locally. This would be equivalent of an ambulatory outpatients visit in which similar assessments are performed. They generally require 30–60 min of work on behalf of the clinician and of the case manager.

Early in the pandemic the local number of visits to ambulatory care practices declined by nearly 60%, in the second pandemic wave by nearly 30%.

We reduced to 15% the number of patients with missing appointments by performing videocall consultations.

### Intermediate level

In the intermediate level, mere videocalls are not sufficient both for the number of specialists involved and to the introduction of additional tools. Here, teleconsultations occur with specialists in a multidisciplinary context, also using several aids like questionnaires or video-tutorials to engage carers to perform simple procedures to assess aspects that are relevant for a decision making process. Carers may also be required to send specific clinical parameters, such as height or weight, after being taught to assess them in a standardized reproducible manner. This would be the equivalent of a Day Hospital setting and take up to 240 min.

In some cases, for more delicate assessments, when the need for monitoring is a one-off (i.e. pulse oximeter for night oximetry measurements), specific devices can be sent to families and returned to the centre for review and analysis of the results.

We have been able to transition approximately 10% of our multi-disciplinary clinic to a virtual telehealth format, primarily utilizing video visits, facilitating clear communication, and enabling our patients to continue and progress through home-based tutorials and procedures. Parent and patients feedback has been largely positive.

### Intermediate level Plus

The Plus option is reserved for when, in addition to simplified tests and indirect clinical assessments, the caregiver is asked to perform an intervention for the care of specific aspects (i.e. rehabilitative exercises, stretching, gastrostomy and tracheostomy care). Parents/guardians will be specifically trained and required to demonstrate what learned. Parental training through specific video sessions are fundamental to guarantee the success of home-care and must be individualized to the child and family’s resources. The videos for training sessions are selected for each patient from a large library of videos that has been specifically built for this purpose and, when possible, provided beforehand so that carers have time to familiarize with the task required. Only after verifying that parents/guardians have acquired the necessary competences, will they be enabled to perform a guided intervention under the live supervision of the clinician, during the teleconsultation. This service, comparable to Outpatient Day Services or Multiple Assessments by Specialists, should up to 240 min in total.

More than 80% of families enrolled in this level completed treatment, with 92% of core sessions attended. Therapists attained 90% fidelity to the training session.

Preliminary efficacy findings suggest that training via telehealth was acceptable to all parents who felt that this modality helped to reduce the effects related to lack of treatment due to Covid.

### Advanced level

This option covers the broadest range of services with the help of multimodal devices sent to the family that can automatically send parameters to a ‘Listening Centre’ active 24/7. Alerts are also sent off to the hospital in the case of abnormal values and can immediately activate an ambulance and enable access to the Emergency Department. This package is equivalent of Multiple Assessments by Specialists and Emergency Medicine.

A small group of patients [8] with high clinical complexity is still receiving medical assistance by a multi-level device with home monitoring.

It is the responsibility of the physician to establish which package, or combination of packages, among the above (teleconsultation, telemonitoring or tele-intervention) best satisfies each patient’s needs and, in some cases, a ‘mixed circuit’, in which the patient alternates in-hospital visits and Contactless services, will be needed.

Aside from the virtual training sessions, the Units must identify a nurse or case-manager whose duty is to instruct parents/guardians on how to download and use the platform and verify that the conditions for an effective connection have been set-up. The nurse or case manager must make sure that the applicant possesses the technological equipment necessary for the provision of the service and carry out necessary connection tests.

For appropriate traceability and service quality monitoring, all delivered sessions will be regularly registered on FPG-IRCCS’ Pediatric Information System and the Patient Platform will regularly emit questionnaires to be filled in by children’s caregivers, soliciting for assessments regarding physical functionality, quality of life, and surveys to measure satisfaction with the service. The selected KPIs will then be used as Impact Measurement tools to assess impact on care, research, value for patients and families.

Given the multitude of actors involved in the organizational model, to aid the construction of a valid regulatory and operational plan, we established a matrix of activities and responsibilities which follow the specificity of each technological device and software adopted and allows to monitor the activities.

To verify the effects of the introduction of the case manager, we defined a set of key performance indicators, as follows:

- → Percentage of drop-outs from telehealth visits (number of missing telehealth visits by pediatric patients/number of fixed telehealth visits for pediatric patients) compared to the percentage of drop-outs from in-hospital visits.

To evaluate the utilization and the satisfaction of the ‘Contactless’ platform the following key performance indicators will be calculated:

- → Average number of contacts recorded in the reference period: 12 months; this indicator makes it possible to know the volume of accesses (quality index) noted by the introduction of the telehealth service.
- → Percentage of telehealth visits rescheduled as in-hospital visits (telehealth visits rescheduled as in-hospital visits for pediatric patients / number of techno care visits). This indicator makes it possible to measure the appropriateness of the selected setting, relative to the complexity gradient of the service.
- → Patient (and caregiver) satisfaction for the ‘Contactless’ service will be investigated with specific scores.

## Discussion

The development of Contactless started from a few concepts shared among clinicians belonging to different disciplines and caring for children with chronic complex disorders and disabilities in need of regular follow up multidisciplinary assessments. The idea was prompted by a letter from a group of leading families to the Observatory of Rare diseases highlighting the difficulties in maintaining adherence to care recommendations and the lack of infrastructures to assist patients and their families. It was felt that, even if the pandemic had reached its peak and the overall management of children and rehabilitative services was about to restart, returning to a pre-COVID routine was unlikely to happen in the near future and activities easy to be performed at home with limited resources should be strongly encouraged. In children with chronic diseases, this included many activities and procedures aimed at monitoring disease course that often do not need sophisticated equipment or devices such as those used in more acute conditions. At the same time however, it was also felt that these procedures, even if relatively simple, should be performed in a structured systematic manner, using as much as possible similar procedures to those used in a clinical setting. A strong interaction between our medical staff and patient associations helped to identify the families’ primary needs and to select tools to help the families to be actively engaged. Particular attention was paid to identify tools that should be integrated with the videoconsulting sessions that had already been implemented. This included the use of questionnaires that could reliably assess different aspects of care (sleep, nutrition) and the use of video tutorial sessions to enable carers to perform properly at least part of the activities that are routinely performed in clinics. Similarly, video training sessions were recorded for more complex activities that would require a more careful explanation of the procedure, such as performing stretching or other activities that are essential in patients with motor difficulties, or taking care of wounds, tube feeding devices or tracheostomy.

We recorded a large number of videos to be used for different ages and conditions. The key element in our approach is that their use is part of an interactive approach between carers and clinicians. Carers have the chance to consult the videos on our platform and perform the activities ‘live’ with clinicians’ supervision. This approach is based on the evidence that, in pediatric care, the custom is to relate to a very large extent with the child’s parents with an engagement of the entire family. The mutual trust relationship between parents/guardians and doctors can be maintained in this new context as there should be no difference compared to in-hospital visits in the time and energy dedicated to empowering parents in the management of their children.

Our program also includes access to devices that will allow monitoring of cardiac and respiratory function with data transmission. While this is somehow the gold standard of telemedicine, the costs and the availability of devices are a limiting factor for a widespread use and is mainly exclusively devoted to children with more complex clinical conditions.

Establishing the Multidisciplinary and Multiprofessional Team, providing patient platforms to exchange information between patients and their care providers and introducing the figure of the case manager or of a dedicated nurse has been essential to significantly improve patient’s and caregivers’ perception of quality of care and compliance with the therapeutic process with a consequent equitable increase in access to care [8–10].

The preferred methodology for the elaboration and development of the Project was the one suggested by the American Academy of Pediatrics [11] which supported the decision making process in the definition of the flow of actions to be undertaken. One of the strengths of our methodology was that we expanded the concept of Telehealth to aspects of care that are more specific for patients with rare disorders, or other conditions that, even in the absence of acute events, are in need of regular follow up. Another strong point of this project relies on its replicability and in the limited resources needed to have an impact on patients, caregivers and professionals. Certainly, the extension of our model to other patients and clinical settings requires readapting the model according to the specific care setting, available resources and facility under consideration.

## Conclusions

‘Contactless’ is a pilot project aimed at facilitating communication and maintaining standards of care with patients with chronic disabilities and and their carers. It is expected to ease direct costs (i.e. transport, overnight stays) and indirect costs (work and school permits or organizational expenses) for families [12, 13] but comes with costs for the hospital. The needs assessment analysis led to an estimated volume of 1366 patients per month declined according to the complexity gradient of each package of care services. In the light of this, the organizational model provided will now serve a point of departure that will be used to support a preliminary business plan including budget, timeline, goals and possible expected revenues. Due to the complex and dynamic needs of pediatric patients, a reimbursement model is needed to adapt to the emergent needs of the ‘Contactless’ care model (i.e. telehealth services, case manager hiring). In contrast to traditional fee-for-service payments, value-based Bundle (or episode-of-care based) payment could cover all the services of the entire cycle of care.

Requiring the engagement of a multitude of stakeholders, including patients and the public, such a trigger needs a strong local and regional commitment to be introduced into routine clinical practice as it is deemed sustainable also after COVID-19 pandemic [4]. While the current approach is to fully equate remote services to in-hospital visits under all aspects of care [12] we are planning to expand the service to include community services and Local Health Units. A Hub&Spoke model would be an optimal tool to guarantee care continuity, possibly activating links with areas distant from tertiary care centres.

## Supplementary Information

**Additional file 1: Table S1.** Forecasted monthly volumes.

## Data Availability

Not applicable.

## References

[1] Nacoti M, Ciocca M, Giupponi A (2020). At the epicenter of the Covid-19 pandemic and humanitarian crises in Italy: changing perspectives on preparation and mitigation.

[2] De Belvis AG, Morsella A, Pastorino G (2020). European Observatory On Health Care Systems And Policies. Covid 19 Health System Response System.

[3] Viganò M, Mantovani L, Cozzolino P, Harari S. Treat all COVID 19-positive patients, but do not forget those negative with chronic diseases. Intern Emerg Med. 2020:1–4. 10.1007/s11739-020-02395-z. 10.1007/s11739-020-02532-8 PMC7573244 33079357

[4] Rametta SC, Fridinger SE, Gonzalez AK, et al. Analyzing 2,589 child neurology telehealth encounters necessitated by the COVID-19 pandemic. Neurology. 2020. 10.1212/WNL.0000000000010010. 10.1212/WNL.0000000000010010 PMC7538222 32518152

[5] Heymann DL, Shindo N (2020). WHO scientific and technical advisory Group for Infectious Hazards. COVID-19: what is next for public health?. Lancet..

[6] Hong Z, Li N, Li D (2020). Telemedicine during the COVID-19 pandemic: experiences from Western China. J Med Internet Res.

[7] Fernandez-Moyano AV-MI, López-Jimeno W (2018). Telehealth. N Engl J Med.

[8] Winegard B, Miller EG, Slamon NB (2017). Use of Telehealth in pediatric palliative care. Telemed J E Health.

[9] Palmer K, Marengoni A, Forjaz MJ (2018). Multimorbidity care model: recommendations from the consensus meeting of the joint action on chronic diseases and promoting healthy ageing across the life cycle (JA-CHRODIS). Health Policy.

[10] Palmer K, Carfi A, Angioletti C (2019). A methodological approach for implementing an integrated multimorbidity care model: results from the pre-implementation stage of joint action CHRODIS-PLUS. Int J Environ Res Public Health.

[11] Pediatrics AAo. Getting Started in Telehealth [Available from: https://www.aap.org/en-us/professional-resources/practice-transformation/telehealth/Pages/Getting-Started-in-Telehealth.aspx].

[12] Olson CA, McSwain SD, Curfman AL, Chuo J (2018). The current pediatric Telehealth landscape. Pediatrics.

[13] Marcin JP, Shaikh U, Steinhorn RH (2016). Addressing health disparities in rural communities using telehealth. Pediatr Res.

