# Supplementary material for: Contactless: a new personalised telehealth model in chronic pediatric diseases and disability during the COVID-19 era
Source: Ital J Pediatr. 2021 Feb 12;47:29. doi: 10.1186/s13052-021-00975-z (PMC7880513; doi:10.1186/s13052-021-00975-z)
Supplement: Supplementary file 1 — Additional file 1: Table S1. Forecasted monthly volumes. [file 13052_2021_975_MOESM1_ESM.docx]

**Supplementary tables – Forecasted monthly volumes**

1. **Rare diseases**

|  |  | **RARE DISEASES** | |
| --- | --- | --- | --- |
|  |  | FORECASTED MONTHLY VOLUMES | HEALTH SERVICES |
| CONTACTLESS  BASIC |  | 160 | Health status and medical history update Clinical Assessment (Specific topic) Evaluation of laboratory or technical tests carried out outside FPG-IRCCS setting Evaluation of 'red flags' (seizures, psychotic break-downs, breathing difficulties…) Contact with patients in radiologic list |
| CONTACTLESS  INTERMEDIATE |  | 80 | Multisciplinary consultations Video tutorials (auxology, management of PEG, tracheostomies, artificial feeding) |
|  | PLUS | 40 | Meal surveillance Training (management of PEG devices, PEJ, tracheostomies, advanced medication) |
| CONTACTLESS ADVANCED |  | 3 | Pulse oximetry with dispatched devices Discussions about diaries/surveys (sleeping, urination, diet QoL) |

Total monthly volumes for the Unit: 283

1. **Pediatric Oncology**

|  |  | **PEDIATRIC ONCOLOGY** | |
| --- | --- | --- | --- |
|  |  | FORECASTED MONTHLY VOLUMES | HEALTH SERVICES |
| CONTACTLESS  BASIC |  | 40 | Contact with patients in radiologic list  Update of health status Evaluation of laboratory or technical tests carried out outside FPG-IRCCS |
| CONTACTLESS  INTERMEDIATE |  | 80 | Blood test in toxic patients, anemia, trombocytopaenia |
|  | PLUS | 16 | Management of central venous lines Venouswithdrawal from a central venous line Medication |
| CONTACTLESS ADVANCED |  | 6 | Modulation of chemotherapy  Administration of growth factors |

Total monthly volumes for the Unit: 142

1. **Child Neuropsychiatry**

|  |  | **CHILD NEUROPSYCHIATRY** | |
| --- | --- | --- | --- |
|  |  | FORECASTED MONTHLY VOLUMES | HEALTH SERVICES |
| CONTACTLESS  BASIC |  | 120 | Health status and medical history update Clinical Assessment (Specific topic) Evaluation of laboratory or technical tests carried out outside FPG-IRCCS setting Evaluation of 'red flags' (seizures, deterioration) Contact with patients in radiologic list |
| CONTACTLESS  INTERMEDIATE |  | 80 | Multisciplinary consultation Video tutorials (management of epilepsy, management of dystonic reactions) Evaluation of neurological signals |
|  | PLUS | 40 | Training (antiepileptic drugs) |
| CONTACTLESS ADVANCED |  | 10 | Pulse oximetry with dispatched device Diaries/surveys (sleeping, seizures, QoL) |

Total monthly volumes for the Unit: 250

1. **Pediatric Neuromuscular Omnicentre - NEMO**

|  |  | **PEDIATRIC NEUROMUSCOLAR OMNICENTRE - NEMO** | |
| --- | --- | --- | --- |
|  |  | FORECASTED MONTHLY VOLUMES | HEALTH SERVICES |
| CONTACTLESS  BASIC |  | 80 | Health status and medical history update Clinical Assessment (Specific topic) Evaluation of laboratory or technical tests carried out outside FPG-IRCCS setting Evaluation of 'red flags' (desaturation, changes of respiratory patterns |
| CONTACTLESS  INTERMEDIATE |  | 60 | Multisciplinary consultation Video tutorial (CHOP, stretching) |
|  | PLUS | 20 | Observation of meals Training (management of device, cough assist machine, Non Invasive Ventilations) |
| CONTACTLESS ADVANCED |  | 15 | Pulse ox with dispatched device Testing and medical prescriptions of orthosis |

Total monthly volumes for the Unit: 175

1. **Pediatric Surgery**

|  |  | **PEDIATRIC SURGERY** | |
| --- | --- | --- | --- |
|  |  | FORECASTED MONTHLY VOLUMES | HEALTH SERVICES |
| CONTACTLESS  BASIC |  | 120 | Health status and medical history update Evaluation of 'red flags' (effort in urinating) Observation of urine flow Evaluation of genital congenital anomalies |
| CONTACTLESS  INTERMEDIATE |  | 2 | Videotutorial: Substitution of gastrostomy tube Replacement of enterostomy pounches |
|  | PLUS | 0.5 | Training:  Management of device Anal dilatation |
| CONTACTLESS ADVANCED |  | Service not provided | Service not provided |

Total monthly volumes for the Unit: 122

1. **Spina Bifida**

|  |  | **SPINA BIFIDA** | |
| --- | --- | --- | --- |
|  |  | FORECASTED MONTHLY VOLUMES | HEALTH SERVICES |
| CONTACTLESS  BASIC |  | 120 | Health status and medical history update Clinical Assessment (specific topic) Evaluation of 'red flags' (vomit, abdominal distension) Evaluation of laboratory or technical tests carried out outside FPG-IRCCS |
| CONTACTLESS  INTERMEDIATE |  | 80 | Videotutorial  (colic clense, urine collection, bladder catheterization) |
|  | PLUS | 40 | Training (bladder catheterization, bedsores, pelvic floor strengthening) |
| CONTACTLESS ADVANCED |  | 10 | Discussions about diaries/surveys (sleeping, urination, diet, QoL) |

Total monthly volumes per unit: 250

1. **Pediatrics**

|  |  | **PEDIATRICS** | |
| --- | --- | --- | --- |
|  |  | FORECASTED MONTHLY VOLUMES | HEALTH SERVICES |
| CONTACTLESS  BASIC |  | 80 | Health status and medical history update Clinical Assessment (specific topic) Evaluation of 'red flags' (specific to each health need) Evaluation of laboratory or technical tests carried out outside FPG-IRCCS |
| CONTACTLESS  INTERMEDIATE |  | 40 | Multisciplinary consultation Videotutorial (auxology, etc ) |
|  | PLUS | 20 | Training (specific specific to each health need |
| CONTACTLESS ADVANCED |  | 4 | Discussions about diaries/surveys (specific to each health need) |

Total monthly volumes per unit: 144
